# Implementing combined WHO mhGAP and adapted group interpersonal psychotherapy to address depression and mental health needs of pregnant adolescents in Kenyan primary health care settings (INSPIRE): a study protocol for pilot feasibility trial of the integrated intervention in LMIC settings

**DOI:** 10.1186/s40814-020-00652-8

**Published:** 2020-09-22

**Authors:** Manasi Kumar, Keng-Yen Huang, Caleb Othieno, Dalton Wamalwa, Kimberly Hoagwood, Jurgen Unutzer, Shekhar Saxena, Inge Petersen, Simon Njuguna, Beatrice Amugune, Onesmus Gachuno, Fred Ssewamala, Mary McKay

**Affiliations:** 1 Department of Psychiatry, University of Nairobi, Nairobi, Kenya; 2 Department of Population Health, New York University School of Medicine, New York, USA; 3 Department of Pediatrics and Child Health, University of Nairobi, Nairobi, Kenya; 4 Department of Child and Adolescent Psychiatry, NYU Langone Health, New York, USA; 5 Department of Psychiatry, University of Washington, Seattle, USA; 6 Department of Global Health and Population, Chan School of Public Health, Harvard University, Boston, USA; 7 Department of Psychology, University of Kwa-Zulu Natal, Durban, South Africa; 8 Department of Mental Health, Ministry of Health, Nairobi, Kenya; 9 School of Pharmacy, University of Nairobi, Nairobi, Kenya; 10 Department of Obstertrics and Gynacology, University of Nairobi, Nairobi, Kenya; 11 Brown School at Washington University in St.Louis, St. Louis, USA; 12 George Warren Brown School of Social Work, Washington University in St. Louis, St. Louis, USA

**Keywords:** Adolescents, Pregnancy, Perinatal mental health, Depression, WHO mhGAP, Group interpersonal psychotherapy, Intervention implementation, Mental health capacity building

## Abstract

**Background:**

Addressing adolescent pregnancies associated health burden demands new ways of organizing maternal and child mental health services to meet multiple needs of this group. There is a need to strengthen integration of sustainable evidence-based mental health interventions in primary health care settings for pregnant adolescents. The proposed study is guided by implementation science frameworks with key objective of implementing a pilot trial testing a full IPT-G version along with IPT-G mini version under the mhGAP/IPT-G service framework and to study feasibility of the integrated mhGAP/IPT-G adolescent peripartum depression care delivery model and estimate if a low cost and compressed version of IPT-G intervention would result in similar size of effect on mental health and family functioning as the Full IPT-G. There are two sub- studies embedded which are: 1) To identify multi-level system implementation barriers and strategies guided by the Consolidated Framework for Implementation Research (CFIR) to enhance perinatal mhGAP-depression care and evidence-based intervention integration (i.e., group interpersonal psychotherapy; IPT-G) for pregnant adolescents in primary care contexts; 2) To use findings from aim 1 and observational data from Maternal and Child Health (MCH) clinics that run within primary health care facilities to develop a mental health implementation workflow plan that has buy-in from key stakeholders, as well as to develop a modified protocol and implementation training manual for building health facility staff’s capacity in implementing the integrated mhGAP/IPT-G depression care.

**Methods:**

For the primary objective of studying feasibility of the integrated mhGAP/IPT-G depression care in MCH service context for adolescent perinatal depression, we will recruit 90 pregnant adolescents to a three-arm pilot intervention (unmasked) trial study (IPT-G Full, IPT-G Mini, and wait-list control in the context of mhGAP care). Pregnant adolescents ages 13–18, in their 1st-2nd trimester with a depression score of 13 and above on EPDS would be recruited. Proctor’s implementation evaluation model will be used. Feasibility and acceptability of the intervention implementation and size of effects on mental health and family functioning will be estimated using mixed method data collection from caregivers of adolescents, adolescents, and health care providers. In the two sub-studies, stakeholders representing diverse perspectives will be recruited and focus group discussions data will be gathered. For aim 2, to build capacity for mhGAP-approach of adolescent depression care and research, the implementation-capacity training manual will be applied to train 20 providers, 12 IPT-G implementers/health workers and 16 Kenyan researchers. Acceptability and appropriateness of the training approach will be assessed. Additional feedback related to co-located service delivery model, task-shifting and task-sharing approach of IPT-G delivery will be gathered for further manual improvement.

**Discussion:**

This intervention and service design are in line with policy priority of Government of Kenya, Kenya Vision 2030, World Health Organization, and UN Sustainable Development Goals that focus on improving capacity of mental health service systems to reduce maternal, child, adolescent health and mental health disparities in LMICs. Successfully carrying out this study in Kenya will provide an evidence-based intervention service development and implementation model for adolescents in other Sub-Saharan African (SSA) countries. The study is funded by FIC/NIH under K43 grant.

## Background

Globally depression is the leading cause of disease burden in women of reproductive age. Maternal depression occurs in women during both antenatal and postnatal periods. The prevalence of depression is high among pregnant women, with worldwide estimates of 11% to 18% [1, 2] and is estimated to be between 15% to 28% in Lower- and Middle-Income Countries (LMICs) [3]. Studies in Kenya and neighboring countries have found that younger women (aged 18–24 years) experience greater psychological vulnerabilities [4] and depression in this group ranges from 8.3% to 39% [5–7] with high risk populations like adolescent mothers and gender-based violence impacted women [8]. Young women in Sub-Saharan Africa (SSA) lose around 10.4 disability adjusted life years due to depression during pregnancy. Women who experience antepartum depression often continue to experience depressive symptoms into the postpartum period, with more than 54% of those with postpartum depression reporting depressive episodes before or during pregnancy [9]. Pregnant women between ages 18–21 years are at high risk for depression in countries like Kenya because circumstances that lead to their pregnancy often include low socioeconomic status and food insecurity [10], with subsequent increases in unprotected sex with older men in an effort to seek financial support through sexual favors. These economic factors also contribute to early motherhood, gender-based violence, low educational attainment and social stigma, all of which are associated with higher prevalence of depression [8, 11–16]. These vulnerable young women need health care and psychosocial support in building resilience against challenging barriers and risk factors.

Consistent with the World Health Organization (WHO) recommendations for LMICs, we propose Interpersonal Psychotherapy (IPT), an evidence-based depression treatment with demonstrated clinical efficacy in East African regions of Kenya and Uganda, for young women’s depression during pregnancy [17, 18]. IPT has demonstrated high efficacy when delivered by a readily available workforce of local, non-specialist personnel in East Africa [17, 19–22]. Based on recent studies utilizing IPT-G in Kenya [18, 23], we believe that IPT’s focus on social support, rebuilding interpersonal relationships by bolstering interpersonal communications skills, and focusing on life-transitions make it a promising fit with the mental health care needs of pregnant adolescents. However, pregnant adolescents in Kenya experience a particular set of stressors which we believe will require adaptation of standard IPT in order to match this particular population’s needs. For example, pregnant adolescents and adolescent mothers in Kenya are often unable to complete education as planned with related shortcomings in earning potential and are susceptible to exploitation and abuse by their biological families and community in general. While IPT is well-equipped to address most of these issues, some adaptations are necessary to address specific role transitions, adverse life circumstances and losses faced by young pregnant adolescents and women in Kenya.

### Adolescent pregnancies and unmet adolescent needs in Kenya

Adolescents make up nearly 16% of the world’s population, and the proportion is greater in Sub-Saharan Africa (23% of the regional population) [24]. Over 16 million adolescent girls worldwide give birth between ages 15–19, and the vast majority of these births (95%) occur in LMICs [25]. When adolescent girls become mothers, their opportunities for economic and educational growth are compromised. A burgeoning number of adolescent mothers from LMICs are marginalized due to poverty, gender-based traditions or geographical resource scarcity which further limit their access to health resources, information, social protection, and personal development [1, 10, 26–29]. Structural inequalities and adverse circumstances have detrimental impact on adolescent mothers’ mental health and this sets the stage for adverse medical conditions, risky behaviors, and mental illness in adulthood [27, 30]. Estimated prevalence of depression during perinatal period is 11% to 18% worldwide and between 30% to 50% in LMICs [1, 31–33]. The risk for perinatal depression in adolescents is 2 to 9 times higher than adults [2, 31, 33–35]. Despite the enormous mental health needs for adolescent mothers in LMICs, perinatal mental health research has focused on adults and has not considered the unique challenges that adolescent mothers are facing. Most research has also not invested in public health strategies or culturally relevant strategies to empower adolescents and address the mental health resource and service gaps for this population in LMIC settings.

### Global mental health service strategies and agenda for addressing needs in LMIC settings

WHO proposed two comprehensive frameworks for supporting efforts to establish and scale-up mental health services in LMICs. The Comprehensive Mental Health Action Plan (MHAP) 2013–2020 (or the Action Plan) [34] proposes system strengthening in 4 areas to improve population mental health: i) developing effective leadership and governance, ii) providing comprehensive, integrated, and responsive mental health services, iii) implementing strategies for promoting mental health, and iv) strengthening evidence and research for mental health. The Mental Health Gap Action Programme (mhGAP) [35, 36] focuses on ii) and iii) of the MHAP and further provides evidence-based guidelines and tools to support service system setup, delivery, and provision of evidence-based interventions (EBIs) for common mental health disorders in health care settings with limited specialty [37]. MHAP is a World Health Assembly approved policy document while mhGAP Intervention Guidelines (mhGAP-IG) are evidence-based guidelines and supportive technical material. mhGAP material helps member states to achieve the objectives of MHAP. Specifically, mhGAP recommends applications of collaborative, task-shifting, and task-sharing implementation strategies (i.e., considering collaboration between mental health specialists and non-specialist health-care providers and redistribution of clinical tasks from mental health specialists to non-specialized providers) in provision of mental health services. The mhGAP-IG also provides assessment, management clinical decision-making action flowcharts, and recommended EBIs for treating 8 priority mental health conditions (including interventions for depression). Although both MHAP and mhGAP have been applied successfully to address adult depression and other mental disorders in several LMICs [20, 38, 39], application of these frameworks in child and adolescent mental health service and in Kenyan contexts remains in infancy. A handful of depression intervention studies in LMICs that use EBIs recommended in the mhGAP (e.g., Group interpersonal psychotherapy/IPT-G, Thinking Healthy Program, Problem Management Plus) have exclusively either focused on perinatal depression for a wide range of reproductive-aged women [15, 27, 40–44] or interventions for depressed adolescents in non-medical health service settings [17, 45]. Most studies have focused on efficacy of EBIs and have not examined implementation related questions or tested feasibility of mhGAP-IG for peripartum adolescent mental health. More pragmatic research that simultaneously considers effective mental health leadership development, workforce development strategies, EBI implementation strategies, and effectiveness outcomes is sorely needed.

In considering utilizing WHO recommended models to adolescent perinatal depression in Kenya, several other system and service level implementation gaps and adolescent population characteristics need to be considered. Like other LMICs, Kenya has weak child and adolescent mental health system with limited mental health professionals and workers [17]. In tandem with the global agenda, the Kenya Vision 2030 mental health policy program (developed in 2012) brings in major health system reforms aiming at ensuring equity, people centeredness and participatory approach, efficiency, multi-sectoral approach and social accountability in delivery of mental health services [46]. However, progress in actualizing these changes in child mental health policy and service system development remain slow.

In addition to the systemic gaps, other intervention-implementation research gaps exist. First, in *the current mhGAP-IG, evidence-based guidelines and tools for adult depression (including perinatal depression) and child and adolescent depression are separated*, but interventions that integrate both pregnancy and adolescent needs for adolescent perinatal depression are lacking. The adult and child/adolescent versions of mhGAP-depression packages focus on different contents and approaches. For example, in adult depression, mhGAP-IG emphasizes on patient’s individual needs and intervention (i.e., psychoeducation, stress reduction and strengthening social support, promoting improved functioning in daily activities, brief psychological treatment for depression, and pharmacology). For adolescent depression, mhGAP-IG considers critical roles of caregivers, families, and schools/teachers in depression intervention given adolescents’ developmental needs; therefore, it recommends that depression intervention for adolescents must consider both individual and family/community related intervention (i.e., consideration of psychoeducation for the child/adolescent and family, guidance on promoting wellbeing, caregiver support, stressors management, community resources linkage, and collaboration with teachers/schools in managing depression). Although both adult and child/adolescent depression intervention guidelines/tool packages are relevant to adolescent perinatal depression, how these packages should be adapted to effectively address Kenyan perinatal adolescent needs is yet to be systematically studied. *Another gap is the demand-resource gap*, as maternal and child health (MCH) clinics usually serve high volume of pregnant adolescents or young mothers (e.g., 20–30 pregnant adolescents are seen per week [or 960–1440 per year] in each MCH clinic in Nairobi, Kenya). Considering high prevalence of adolescent pregnancy, high risk for depression in this population, and low resource in MCH clinics, utilizing WHO recommended depression EBIs (e.g., IPT-G), which usually require more than 5 intervention sessions, may not be practical in meeting population service demands and needs. Additional cost-effective strategies (such as considering lower dose implying fewer intervention sessions, using group instead of individual approach for intervention, applying low-cost patient/family/community empowerment approaches of service provision) would be needed.

In summary, given varied developmental needs and living context differences (e.g., parent-child co-living, connection with educational institutions) between adults and adolescents, more intervention implementation-effectiveness research for perinatal depression in adolescent population is needed. In addition, given the utility, comprehensiveness, and potential impacts of mhGAP, systematically studying the feasibility of integrating mhGAP in routine MCH care for adolescents with perinatal depression merits further inquiry. These areas of research will provide knowledge for providing integrated intervention (or co-located MCH and mental health services) to reduce burden of perinatal depression for adolescents in Kenya and LMIC in general.

**Fig. 1:**
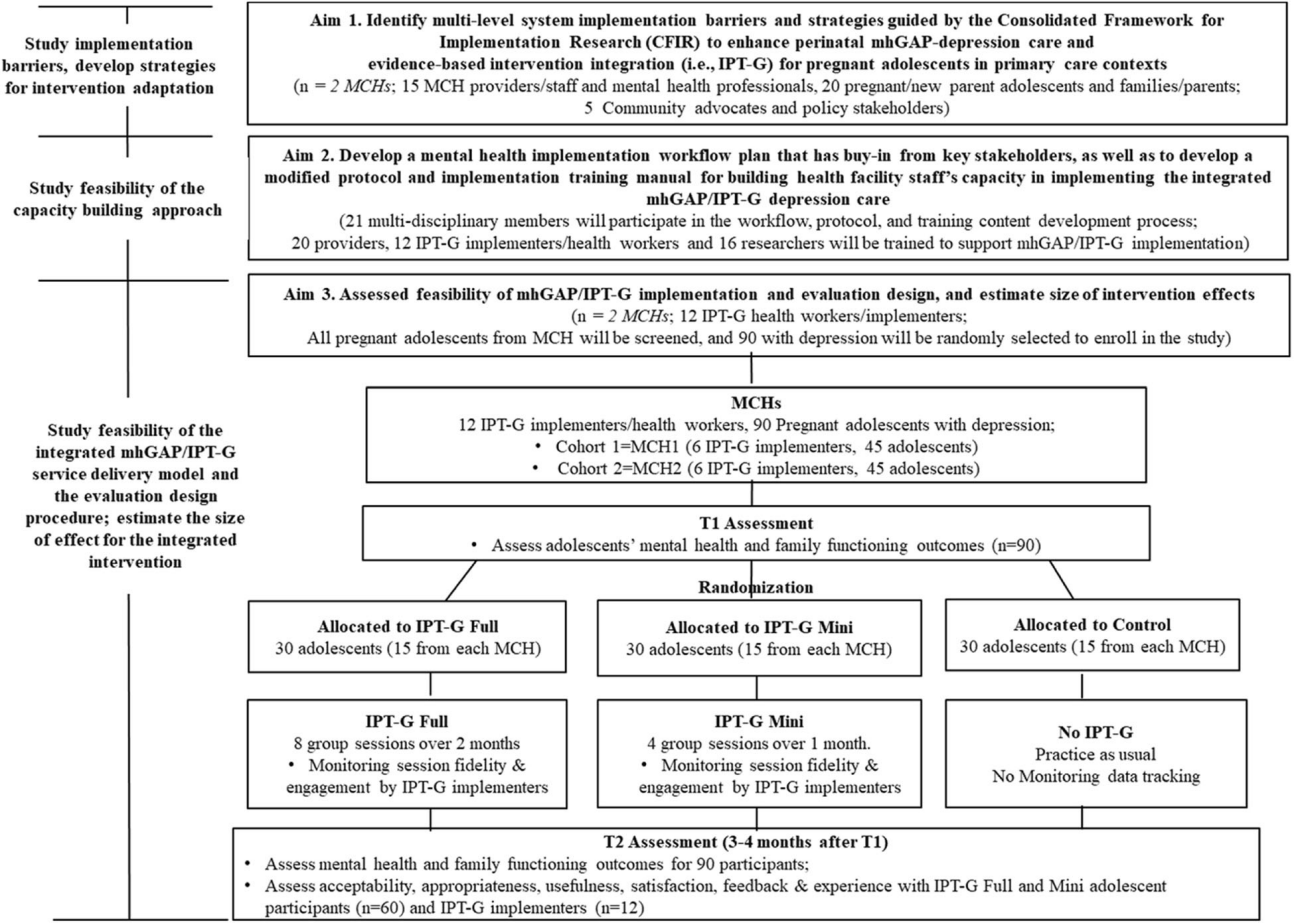
CONSORT for mhGAP/IPT-G Randomized Pilot Feasibility Study

Given this background, the purpose of this study is to identify strategies to improve integration and fit of the mhGAP-depression intervention model (including IPT-G) for pregnant adolescents in Kenya; to develop a Kenyan-adolescent version of WHO recommended mhGAP/IPT-G intervention program and training manual for intervention implementation and service system capacity building; and to pilot test feasibility of this integrated mhGAP/IPT-G service delivery model for adolescent prenatal depression (see Fig. 1 and Tables 1, 2, 3).

**Table 1:** Summary table the pilot feasibility trial

Objectives	Outcomes	Criterion for success	Method for analysis	Estimate of effect
Primary feasibility	Demonstrate that the method of RCT feasible in MCH clinical service context	70% of pregnant adolescent-caregiver-health care workers that are exposed to content of the intervention; 80% of data collection performed as planned;	Quantitative analysis for implementation outcomes will be based on data collected throughout the implementation period, and analyzed separately for each time point as well as jointly to create overall summary scores. Analysis for the MCH service outcomes will be based on pre-post intervention data using pair-t tests and repeated measures. Quantitative data for implementation outcomes, MCH service outcomes, and adolescent mental health effectiveness outcomes will be examined separately. Qualitative data will be transcribed and analyzed using similar techniques described in Aim 1. We will review all focus group discussion data and use grounded theory approach to search for emerging themes using open and axial coding.	Depression and family functioning will be based on multi-level modeling, adjusting for family/group nesting effects. Post-intervention effects estimated as a function of baseline levels of the corresponding outcome variables and intervention status.
Secondary outcomes	a) Identification of barriers and facilitators to mhGAP/IPT-G implementation in MCH context b) Capacity building of MCH personnel, researchers and select MOH and County health partners c) Adaptation of mhGAP/IPT-G	a. Identification of select mhGAP informed barriers to care and adaptation of mhGAP and IPT-G for this context b. Capacity building will significantly improve trainees’ (nurses and community health workers) knowledge and skills in implementation research and delivering of depression intervention. c. Adapted manual for IPT-G and algorithm for mhGAP-IG for depression for pregnant adolescents	Qualitative and ethnographic methods including mixed methods using D& I and attitudes to EBI and adolescent friendly health services Pre and post training interviews and acceptability and satisfaction the trainings Knowledge and fidelity trainings on mhGAP/IPT-G	N/A

**Table 2:** Study measures for the feasibility evaluation study: constructs, informants, assessment schedule, and assessment tools (to accompany the SPIRIT figure)

Constructs, informant, & assessment time	Assessment tools/measures
**Multi-level D&I Context (domains listed in** Fig. 1**)** [Time 1]	
**Inner setting &Individual Characteristics (MCH staff, Adolescent reports)):** * MCH system characteristics; * Implementer demo & attitude about the EBI; * Family characteristics	Provider Environment Questionnaire Survey [45, 47]; Organization climate/Readiness Questionnaire [47]; EBP Attitude Scale [46]; Family Demographics /Social determinants
**mhGAP/IPT-G Implementation Feasibility Outcomes** [Assessed Throughout Implementation Period]	
**a) Fidelity (Staff):** Feasibility in implementing mhGAP service delivery model and delivering > 80% of session content in MCH setting; **b) Engagement (staff report)**: Feasibility in reaching the targeted population (adolescent-parent pairs), and maintain high program participation/session attendance; **c) Quality of Implementation (adolescent-report)**: *Acceptability, Appropriateness Usefulness, Satisfaction (of IPT-G implementation procedures and contents);	**a**: Fidelity Checklists **b:** Program Engagement, attendance; **c.** Acceptability, Appropriateness, Usefulness, Satisfaction;
**MCH Mental Health Service Outcomes** [Time 1, Time 2]	
**a) MCH service quality (adolescent mothers):** patient satisfaction of the integrated mental health & MCH care**;** **b) MCH climate (staff & adolescent report)**: support, trust, communication about adolescent health; responsive adolescent services.	**a:** Service quality; **b:** Organization Climate Questionnaire [47]
**mhGAP/IPT-G trial design feasibility and effect size estimation for mental health & family functioning outcomes** [Time 1, Time 2]	
a) **Design feasibility:** is the method of RCT feasible in MCH clinical service context **b) Estimated size of effect on adolescent mental health (adolescent report);** **c) Estimated size of effect on family functioning (adolescent & caregiver report):** functioning, depression, trauma, adjustment, social support, self-efficacy, stress, family interpersonal communication.	**a:** % of adolescent-caregiver pairs that are exposed to content of the intervention; % of data collection performed as planned; **b**:WHO-Disability Assessment Schedule; Edinburgh Postpartum Depression Scale; Patient Health Questionnaire-9;**c:** Self-efficacy; Social support; PTSD Patient Checklist-Civilian; Kessler Psychological Distress Scale; Adolescent Interpersonal Connectedness and Conflict Inventory [48–55]

Note. The primary outcomes for this pilot feasibility study are mhGAP/IPT-G effectiveness outcomes

Implementation and service outcomes are the intermediate or secondary outcomes of this study

**Table 3:** Information about the technical and community advisory boards

Community Advisory Board will include the following representatives: − 6 MCH (2 leader/directors, 2 nurse, 2 CHW) − 2 governmental (1 MoE, 1 MoH) − 2 academics (1 health service/implementation, 1 clinical researcher) − 2 advocacy (2 representative NGO leader) − 5 community stakeholders (2 caregivers of pregnant adolescent, 3 adolescent representatives). The technical advisory board comprises of following members: -UNFPA program officer (responsible for sexual and reproductive health issues, working on adolescent girl child mental health. - Director, mental health services, MoH (responsible for integrating mhGAP in clinical workflow at primary care level) - Research officer, Nairobi County Health Directorate (Responsible for task sharing and stigma reduction strategies to address depression) -WHO, Kenya county office -Mental health focal officer (Responsible WHO mhGAP adaptation and local capacity building in mental health) -WHO HQ Geneva (Responsible for G-IPT adaptation and mhGAP for adolescent peripartum depression management) -UNICEF Regional office- MCH and adolescent health program officer advise on MCH and adolescent mental health policy and programs)	

The adolescent version of mhGAP/IPT-G is yet to be developed in Kenya. We are proposing a pilot study to test feasibility of the trial study design and estimate size of mhGAP/IPT-G intervention effects (Full and Mini versions) on pregnant adolescents’ mental health and family functioning.

As a first step, we will bring a group of leaders/stakeholders from MCHs and communities together to focus on:

1. strategies to enhance the fit of mhGAP/IPT-G into Kenyan pregnant and parenting adolescents’ needs as well as MCH clinical structure, resources, and workflow (in addition to the task-shifting/sharing and collaborative strategies that have been included as part of the mhGAP);
2. strategies for designing adolescent version of mhGAP/IPT-G training manual and effective capacity building approach to support the implementation of the integrated mhGAP/IPT-G service delivery in MCHs.

## Conceptual model and design overview

### Conceptual model

The adaptation, implementation, and evaluation of the mhGAP/IPT-G will be guided by an integrated conceptual model that combines two dissemination and implementation (D&I) frameworks. D&I research is a growing field that informs how evidence-based interventions and practices can be successfully adopted, implemented, and maintained in health care delivery and community settings. Its methods and frameworks provide guidance around building teams, engagement with stakeholders, maintaining fidelity and adapting tools and interventions to varied cultural and organizational settings [48]. The *Consolidated Framework for Implementation Research (CFIR)* [48, 49], which accounts for five domains of contextual factors that may influence key determinants that impact implementation and effectiveness outcomes, will be used to guide implementation and adaptation strategy development under aims 1 & 2. The five major domains of the CFIR include the *intervention, individuals involved*, *the inner* (including political, structural, and cultural contexts) and *outer* (including social, economic, and political contexts) *settings*, and *the process by which implementation is accomplished*. The *Conceptual model of Implementation Outcomes* [56], which provides a framework for guiding implementation, service feasibility, and effect size/impact estimation, will be applied to guide the aim 3 outcome estimation. Applying both multi-domain/level D&I frameworks will result in: (1) improved strategies to address knowledge gaps toward adolescent friendly and family-centered adolescent pregnancy and mental health care; (2) identification of low-cost support strategies from health care workers, family members and community to support the adolescent; (3) engaged/motivated strategies for adolescents toward making proactive behavioral and health changes (i.e., for promoting their reproductive health and wellbeing); (4) integrated quality MCH services supported by local providers and policy stakeholders; and (5) evidence for effective mhGAP/IPT-G implementation and potential efficacy outcomes.

### Design overview and setting of the study

A rigorous mixed methods study will be used to study strategies to adapt and improve mhGAP-IG and IPT-G for Kenyan adolescent population and MCH implementation contexts. The pilot implementation study will be based at two Nairobi primary health care centers—Kariobangi North and Kangemi. Kariobangi is a low-income residential estate in north eastern part of Nairobi, Kenya. Kariobangi consists of both lower middle class and slum-type dwellings and is split into two parts: Kariobangi North and Kariobangi South. Kariobangi North is located approximately 15 kms from Nairobi city center. Kariobangi north is estimated to have 8000 to 10,000 residents. Kangemi is a slum in Nairobi County located in a small valley on the outskirts of the city. It is bordered on the north by the middle-class neighborhoods of Loresho and Kibagare and Westlands on its west. Different sources report that Kangemi has more than 100,000 residents. The study will be conducted in Nairobi at the Nairobi County’s two primary health care facilities that offer MCH services. The study is conducted in collaboration among partners from the University of Nairobi, Ministry of Health (Community Health Promotion and Mental Health Departments), WHO non-communicable diseases unit, and UNFPA’s adolescent SRH section. After enrolment, adolescents will be followed through regular clinic visits in the two respective centers. The study clinic will have dedicated staff including clinical and nursing officers able to provide clinical care offered as part of routine Antenatal Clinic (ANC) care for pregnant adolescents. The assumption is that the pregnant adolescent will seek the intervention and ANC at the clinic site from where she was recruited. The delivery options might vary depending on the personal situation, obstetric history and medical complications associated with pregnancy. However, the participants will be tracked on the delivery and birth outcomes wherever childbirth takes place.

Kenyatta National Hospital/University of Nairobi ethics review committee has approved the study and additional approvals were sought from Nairobi county health directorate and National Commission for Science, Technology and Innovation which will be renewed annually (as per the respective committee policies).

## Methodology

Figure 1 provides an overview of the study procedure and design for the pilot randomization trial. Below we provide detailed methodological approach for the pilot feasibility trial and the two sub-studies.

### Methodology for primary pilot trial

Considering high prevalence of adolescent pregnancy, high risk for depression in this population, and low resource in MCH clinics, the WHO recommended Full version of IPT-G may not be practical in meeting population service demands and needs. Therefore, we will test a Mini version of IPT-G in comparing to the Full version of IPT-G in the context of mhGAP mental health service delivery model. For this Aim, our primary goal is to evaluate the feasibility of implementing the integrated mhGAP-IG/IPT-G service model in the MCH contexts as well as feasibility of applying RCT design to study differential impacts of the IPT-G Full and Mini version in MCHs. Our secondary goal is to estimate the sizes of effect for the mhGAP/IPT-G Full and Mini versions.

#### Study procedure

Figure 1 provides an overview of the pilot trial design. The two MCH clinics that we build capacity in Aim 2 will be the pilot Implementation sites. During the 1st implementation cycle (about 2–3 months), we will work with only 1 MCH clinic (Cohort 1), and then work with another MCH clinic in the 2nd implementation (Cohort 2). For each cohort, 45 pregnant adolescents with moderate to severe depression will be recruited. As the interventions will be using self-reported measures and the adolescent participants would be directly involved the intervention arms would be unmasked.

#### Inclusion criteria (for the implementation feasibility and impact estimate study)

Pregnant adolescents (ages 13–18), screened with moderate to severe depression during the 1st to 2nd trimester (defined as Edinburgh Postnatal Depression Screen (EPDS) score ≥ 13 or meet the APA’s Diagnostic and Statistical Manual for Mental Disorders IV-TR version (DSM-IV-TR) criteria for major depression disorder and perinatal depression), who agree to give informed consent (by adolescents and their adult caregivers), are willing to come for group sessions at MCH clinic, can participate in evaluation assessment and give consent to publish the findings of the work, will be eligible. Participants will be excluded from the study if they suffered from or showed evidence of severe personality disorder, acute psychosis, suicidality, (however will be given referral to appropriate services upon identification of any significant risk) or where there will be significant substance abuse, or evidence of severe comorbidities.

#### Intervention implementation

Participants will be randomly assigned to IPT-G Full, IPT-G Mini, and wait-list control using computer random generator by research staff (*n* = 15 for each arm, 45 per cohort).

**Fig. 2:**
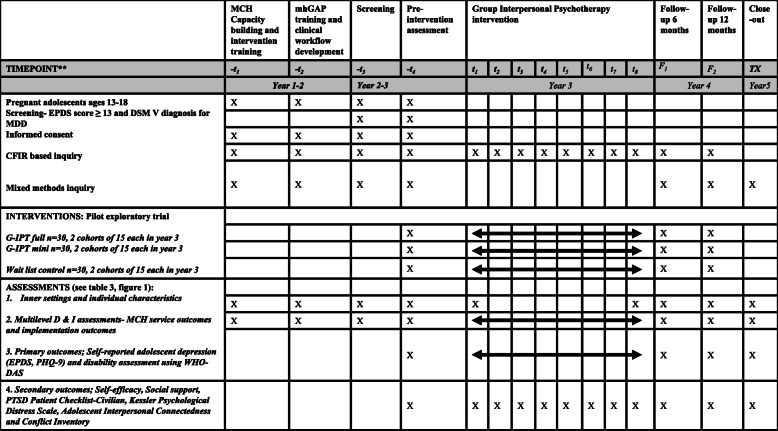
Schedule of Enrolment, Interventions, and Assessments.**Recommended content can be displayed using various schematic formats. See SPIRIT 2013 Explanation and Elaboration for examples from protocols

- The IPT-G Full and Mini groups will receive the assigned interventions (8 vs. 4 sessions) developed from Aims 1 and 2 (including a perinatal depression educational material/handout). To avoid contamination, intervention for the IPT-G Full and Mini versions will be carried out by 2 separate implementation teams (2–3 facilitators in each team) follow the assigned manual/protocol. Figure 2 provides intervention contents for the Mini version, in comparison to the recommended Full version.
- The Control will receive standard care (with referral) and perinatal depression educational materials for safety concerns. They will receive IPT-G Mini after the post implementation assessment is completed.

#### Feasibility evaluation measures and procedure

To evaluate feasibility of the mhGAP-IG/IPT-G implementation model in the MCHs, a mixed methods data collection approach will be applied and data will be collected from multiple sources (i.e., implementers, MCH leaders, adolescent mothers and their family members) to minimize data collection bias. As described above, the *Conceptual model of Implementation Outcomes* [56] is applied to guide the construct selection for the implementation feasibility and impact estimation. Feasibility of the mhGAP/IPT-G mental health service delivery model will be assessed using three indicators: fidelity (i.e., % of sessions is delivered with > 80% of contents), level of engagement (e.g., % reach targeted population, % participating in intervention sessions), quality of implementation (e.g., acceptability, appropriateness, usefulness, satisfaction of the mhGAP/IPT-G service model), and perceived mental health service (e.g., mental health service quality, MCH climate). Feasibility of applying a trial design for mhGAP/IPT-G impact evaluation in MCHs will be assessed using two indicators: % of adolescent-caregiver pairs that are exposed to intervention contents within the assigned condition; and % of data collection performed as planned. Impacts/effect size estimation for the IPT-G Full and Mini versions (the secondary outcomes) will be assessed for adolescents’ mental health and family functioning outcomes. Both quantitative data and qualitative data will be collected. Qualitative data will be collected through focus groups (2 groups for each intervention arm) at the end of implementation cycle. Tables 1 and 4 lists the feasibility outcomes measures and sources of data collection (under implementation feasibility, mental health service outcomes, and trial design feasibility and effect size estimation). Implementation feasibility outcome data, collected from the MCH staff/intervention implementers and adolescents who participated in the two intervention arms (IPT-G Full and Mini), will be gathered after each group session throughout the two implementation periods. For effect-size estimation, all study participants will complete a baseline (Time 1) interview (on mental health and family functioning outcomes) before randomization and intervention implementation, followed by a 1- or 2-month implementation period (2 months for the IPT-G Full and 1 month for the IPT-G Mini). Only when a participant is physically unwell, or has to terminate due to a pregnancy related emergency or any other severe mental health issue will their participant grouping be unblinded and an appropriate referral made. They will receive a post-intervention interview assessment at the end of implementation cycle (Time 2, about 3–4 months after the Time 1). To facilitate coordination of program implementation activities, participating MCH will be provided with two tablets and a small incentive which could offset implementation costs.

**Table 4:** Research implementation steps and data gathering activities

**Research Implementation steps and data gathering activities**	Protocol development, pre-testing of tools and training; formation of technical and community advisory boards	Mixed qualitative methods inquiry on facilitators and barriers to uptake of evidence- based interventions for depression care in primary care settings for pregnant adolescents	Adaptation of mhGAP depression care guidelines and treatment intervention for pregnant adolescents	Observational notes for clinical workflow in primary care and training in integrated mhGAP and IPT-G for depression care	Adaptation of brief IPT-G	Test feasibility of implementation and estimate the size of effect on mental health for the adapted version of mhGAP-IG/ IPT-G.	Analyses, writing and dissemination
**Sample**	Community advisory (17members), Technical advisory board (5 members)	40 participants (8 FGDs with 5 members per group),	Utilizing community advisory and technical advisory boards for adaptation process; expert consultation as and where needed	20 researchers and 16 providers	Utilizing community advisory and technical advisory boards for adaptation process; expert consultation as and where needed	90 pregnant adolescents in a three-arm study: IPT-G Full and IPT-G Mini	Compiling all the information derived from all participants and consultations with mentors, experts and advisory board members
**Location of activity**	In person and virtual meetings with study team members and mentors	Community and health facility based	Desk-based activity including expert consultation with partners from Department of Psychiatry University of Nairobi, Department of Mental Health MoH, WHO, UNFPA and Nairobi county	Community and health facility sample	Community and health facility sample	Community and health facility -based purposive sampling	Virtual, in-person one-on-one and group discussions and meetings with research team, advisory board members and mentors
**Training of health workers and capacity building activities**	Train two-four career researchers, engage communities and build networks with Ministry of Health, mapping a referrals process for any medical or psychosocial issue arising during study	Train data collectors & RAs in two sites, train teams in psychological first aid, identify community health workers for linkages in case of any referrals for those with high depression or at risk for mental or physical	Collaborative work with the research team; three early career researchers and postgraduate students trained in mhGAP and IPT-G; technical and community advisory	Collaborative work with research team, trained researchers to train health facility workers and community health workers; capacity building of the health facility nurses in depression screening for adolescents especially peripartum	Collaborative work with the research team; trained health facility and community providers; technical and community advisory and mentors	Trained data collectors from Aim 1 would be re-engaged and additional trainings and capacity building on survey data collection will be carried out before the survey is collected. Quality assurance checks will be carried out after first 5 participant survey data per site and training repeated if there are discrepancies	Dissemination of findings to Ministry of Health and Nairobi County, Training programs for health facility workers with county and ministry of health using mhGAP
**Data collection method**	Iteratively developed mixed methods inquiry with expert consultation, community advisory support	a. FGDs, KIIs b. Short survey for conjoint experiment	a. Stakeholder engagement through advisory board meetings and group discussions, b. Expert feedback	a. ethnographic- observational study, b. training workshops with the researchers, c. training workshops in the health facilities	a. Stakeholder engagement through advisory board meetings and group discussions, b. Expert feedback	feasibility trial with individual level randomization- Intervention will be delivered in groups at the health facility and data would be collected during each session and after session	Mixed methods analysis: psychometrics/multivariate analyses/ mixed qual methods
**Domains addressed /focal activities**	Comprehensive community based participatory approach integrating strengths and needs of key partners from Ministry of Health, Nairobi County, WHO and UNFPA	Depression care needs from multiple stakeholders; depression care treatment preferences	Adaptations and modifications for WHO mhGAP depression treatment manual and IPT-G manual for peripartum adolescents	Integrated work-flow plan to address mental health needs of pregnant adolescents	Brief version of IPT-G which has been validated for use by technical and community advisory	Multilevel assessments from participants, providers and facility managers on acceptability, appropriateness, and usefulness of the adapted version and implementation process of the mhGAP-IG/ IPT-G service model; and adolescent self- report depression and functioning outcomes;	Several planned publications including: a. mhGAP/IPT-G adaptation for adolescent mothers with depression; b. Acceptability and Appropriateness of the Brief and Long versions of IPT-G intervention implemented by CHWs (results from a mixed method study), c. D&I context measures validation paper, d. Implementation Effectiveness Evaluation (using RCT implementation study data to study effectiveness for brief and long version of IPT-G)
**Outcome measure/s or final output**	Finalized protocol with all IRB clearances and peer reviewed by a wide group of specialists	Multi-stakeholder appraisal of depression care treatment preferences	Integrated mhGAP depression care with modified IPT-G manual	New workflow plan to organize clinical services around mental health care for pregnant adolescents	Finalized version of the manual	Acceptable and useful integrated mhGAP depression care and modified IPT-G for adolescents, health facility and community health workers. Evidence of small to moderate size of intervention effect on adolescents. See Tables 1 and 2	Peer reviewed publications, conference presentations, dissemination in the three communities through Ministry of Health and in the scientific community

#### Sample size

A randomly selected sample of 90 adolescent-parent dyads will be recruited. It has been suggested that a minimum of 30 participants per group/arm is sufficient for individual IPT and IPT-G pilot studies based on similar intervention studies in LMICs [17, 18, 23].

#### Analysis plan for the feasibility outcomes

Data collected through 2 cohorts will be analyzed. Quantitative analysis will be focused on examining the following questions:

- Examine acceptability, appropriateness, usefulness, engagement, and fidelity for both full and mini versions of integrated mhGAP/IPT-G intervention implementation using task-shifting/sharing approach in MCH contexts.
- Examine MCH service quality change patterns before and after implementation of the mhGAP/IPT-G model will improve.
- Examine whether pregnant adolescents in the IPT-G will have better outcomes relative to control, but high impacts/effect size for the IPT-G Full version.

The following stop-go criteria will be used to determine whether to proceed to the main trial: the recruitment proceeds well so that the first IPT-G cohort can proceed by the end of the first month of the specified recruitment period. The IPT therapists that is the trained CHVs and nurses demonstrate adequate levels of treatment fidelity to the WHO IPT-G checklist adherence available in the manual and 70% of participants attend at least 5 of the available sessions in the course of their treatment and 80% are successfully followed up at primary endpoint.

Quantitative analysis for *implementation outcomes* will be based on data collected throughout the implementation period, and analyzed separately for each time point as well as jointly to create overall summary scores. Analysis for the *MCH service outcomes* will be based on pre-post intervention data using *paired-t tests* and repeated measures. We will examine patterns separately and jointly for two MCH clinics. Analysis for *estimating size of impact* on mental health (depression) and family functioning will be based on multi-level modeling, adjusting for family/group nesting effects. Post-intervention effects/impacts will be estimated as a function of baseline levels of the corresponding outcome variables and intervention status. Effect size (regardless the statistical testing significant level) will be computed. *Quantitative data* for implementation outcomes, MCH service outcomes, and adolescent mental health effectiveness outcomes will be examined separately.

*Qualitative data* will be transcribed and analyzed using similar techniques described in Aim 1. We will review all focus group discussion data and use grounded theory approach to search for emerging themes using open and axial coding. Analysis will focus on themes related to MCH service quality (on providing both MCH and mental health care), feedback on implementation process and intervention contents, and perspectives on theory of change.

### Methodology for sub-study 1

**Fig. 3:**
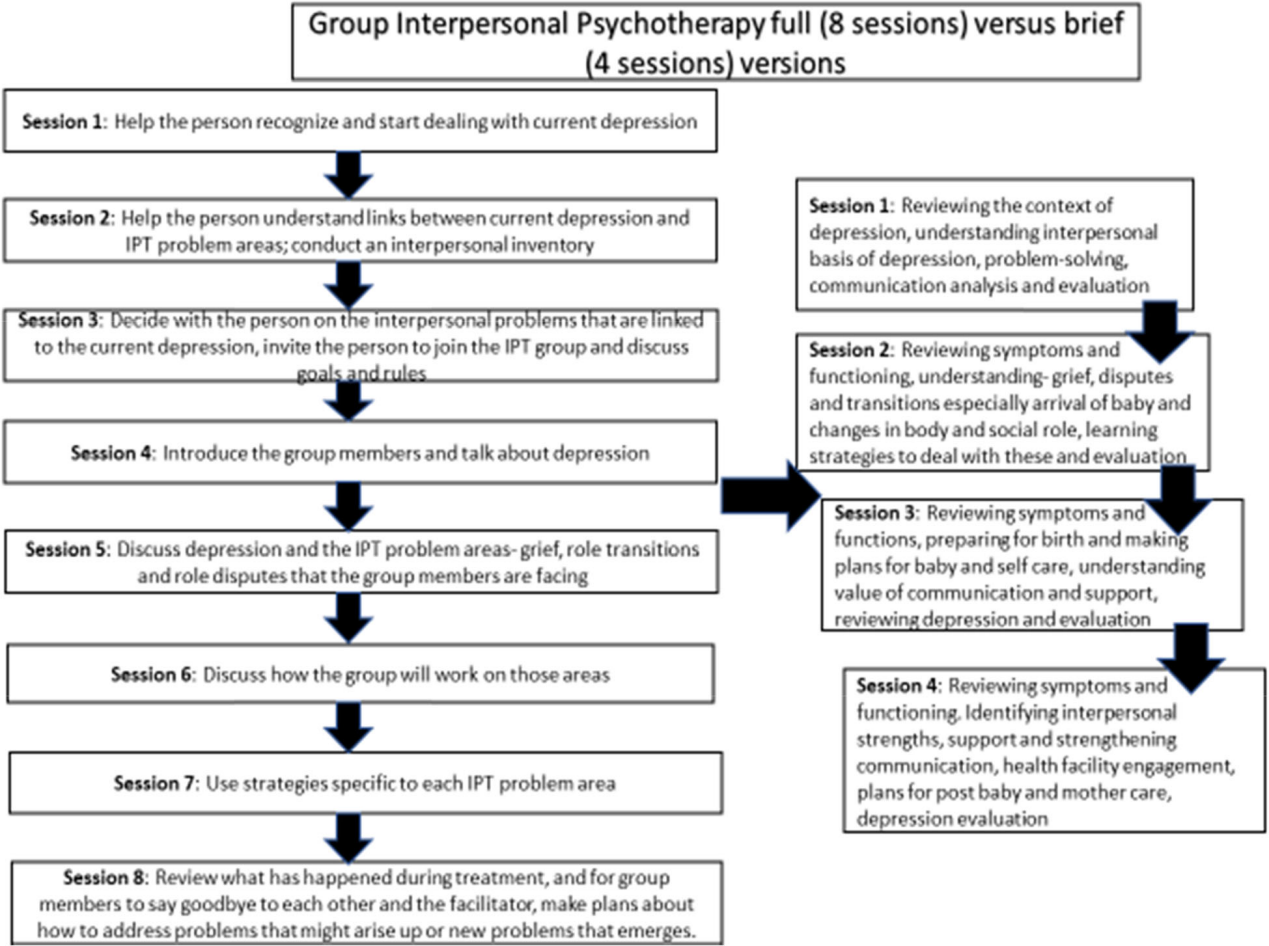
Group Interpersonal Psychotherapy full versus mini (or brief) versions

To understand ‘fit’ of the mhGAP/IPT-G and study barriers/strategies (in 5 CFIR domains) to promote utilization and impacts of mhGAP/IPT-G, we will carry out a mixed methods study, including qualitative and quantitative data collection. Quantitative data collection will focus on D&I readiness contexts and new D&I tools pilot testing (see Table 4 and Fig. 3 for data gathering activities for the entire study and SPIRIT chart).

#### Data collection focus and procedures

We will conduct eight focus groups (*n* = 40) with representative diverse stakeholders (i.e., including two groups for pregnant adolescents and new adolescent mothers, two groups for adolescent caregivers and partners, two groups for MCH staff, 1 group for community advocates and policy leaders, and 1 group for child and adolescent mental health professionals). Each focus group discussion (FGD) will have about 5 participants. Each group will participate in three FGD meetings. One meeting will focus on barriers/strategies related to mhGAP-IG, and the other meeting will focus on barriers/strategies related to IPT-G. The third FGD would be focusing on mental health treatment preferences of pregnant adolescents. Each focus group will last 1½ to 2 h. Participants will participate in an FGD and complete a survey, and these will be carried out in English or Kiswahili (as English is the official language in Kenya). Focus group guides/questions and quantitative survey will be adapted from existing D&I studies [48], or existing D&I measures (see measures under sub-study 1). Stakeholders’ views on barriers and strategies in five CFIR domains will be discussed separately for mhGAP and IPT-G. Informed by our previous needs’ assessment work, strategies related to livelihood problem, partner/family engagement, knowledge gap, and stigma reduction will also be explored. Key information that we will gather from different groups of stakeholders is summarized below.

- *For FGDs with adolescents and families/partners* (*n* = 20, recruited through two MCH clinics that we have partnership with from previous MCH epidemiological studies), discussion will focus on CFIR individual, intervention, and inner setting domains, and examine their knowledge related to perinatal depression, and views (including barriers and potential strategies) related to adolescent pregnancy, mhGAP/IPT-G (fit, usefulness, perceived feasibility and usefulness of IPT-G Full and Mini versions), family member/partner engagement, support, and empowerment strategies, and preference for integrated mental health-MCH services. *Eligibility criteria for adolescent participants* would be: participants of ages 13–18 years, willingness to sign consent form, being pregnant or a new mother for female participants, willing to fill the questionnaires and participate in the interviews, consent to publish the findings. For other stakeholders including health facility providers, adult caregivers, community health workers eligibility includes: above 18 years of age, willingness to sign informed consent to participate in the study and for the deliberations to be recorded, willing to engage with the issues around mental health and adolescent pregnancy conducted through survey or interviews, and consent to publish the findings.
- *For FGDs with MCH providers/staff and mental health professionals* (*n* = 15, recruited through the two MCH clinics [same as the sites for family recruitment] and mental health professional networks), discussion will focus on the CFIR inner setting, individual/staff, intervention, and process domains. We will examine fit, barriers, and implementation strategies for mhGAP and IPT-G implementation in MCH settings (e.g., collaborators’ roles, task-shifting/sharing strategies, workflow logistic/coordination strategies), staff attitude/belief toward EBI practices, perceived effectiveness, and MCH implementation climate.
- *For FGDs with the community advocates and policy stakeholders* (*n* = 5, recruited through Ministry of Health and NGO networks), discussion will focus on the CFIR intervention, process and outer setting domains, and examine barriers and strategies related to mhGAP and IPT-G. In addition, community awareness/public educational and stigma reduction strategies (outer setting strategies) will be explored to facilitate community support to address adolescent pregnancy and maternal mental health needs, which also has implications for public health impact promotion [49, 50].

Results from this study will be used to inform the adaptation of mhGAP and IPT-G contents, adolescents/ family members’ engagement activities, implementation process, and training of the implementation team. An adapted manual for mhGAP-IG and IPT-G (Full and Mini versions) will be developed.

### Methodology for sub-study 2

To better identify mhGAP/IPT-G workflow barriers and strategies in MCH contexts, MCH site visits and workflow observation will be arranged for the Community and Technical Advisory Board members (*n* = 22) (see Table 3). The observation will focus on MCH structure and perinatal service workflow. For the four adolescent representatives, we will seek two pregnant adolescents and two new adolescent mothers. The eligibility criterion would be as follows: willing to consent to participate, one pregnant adolescent and one new mother between ages 13–16, another pregnant adolescent, and new mother between ages 16–18 willing to join the meetings held at the community center or at the health facility. For all other stakeholders of the community advisory, the eligibility would be as follows: to sign informed consent to participate in the study and for the deliberations to be recorded, willing to engage with the issues around mental health and adolescent pregnancy, and willing to commute to the health facility or community center for meetings three times a year. Additionally, there would be a technical advisory board. The technical advisory board comprises of members whose organizations provided support letters during the grant and formally committed to providing technical guidance. Their eligibility includes the following: adults above 18 of years and technical persons representing mental health and health policy implementing partners in Kenya, willing to sign consent form for participation in the study, and willingness for discussion to be recorded. This will allow the Board and research members to gain better understanding about the clinical set up, implementation barriers and strategies, which will facilitate better and more sustainable workflow plan/strategy development to support mhGAP/IPT-G implementation. To ensure confidentiality, informed consent for all participants will be obtained prior to the data collection. Participating provider personnel and family members will be compensated for their time. Participating mental health professionals and officers of NGO/ Ministry of Education/ Ministry of Health will also be compensated.

Results from sub-studies 1 and 2 will be used to inform the adaptation of mhGAP/IPT-G implementation protocol in MCH context and training manual development for mhGAP/IPT-G implementation. Additional feedback will be sought from Advisory Board before using these.

#### Capacity building

*To build implementation research capacity,* the lead investigator along with her co-investigators and mentors will provide a short/intensive 3-day course on child mental health implementation methodology for research team members, relevant stakeholders, and graduate level students (*n* = 20 in year 2). *To build service capacity* for adolescent perinatal depression, we will train MCH implementation team (*n* = 16 from two MCH clinics) for task-shifting/sharing collaborative approach of mhGAP-IG and IPT-G implementation. Members of the implementation team (to be determined in sub-study 1, which may include family members/adolescents if peer leader/empowerment implementation strategy will be used) will receive a 4-day training (including one-day practice training) to prepare them for the implementation. Additional 8-group coaching/support from the clinical team (provided after each IPT-G group session) during the first intervention implementation period will also be provided. The coaching support after the first implementation will be based on the identified needs.

#### Data collection measures and procedures for capacity building

Quality and impacts of training will be assessed through attendance tracking, after training satisfaction evaluation, pre- to post-training knowledge/competence assessment with trainees. The measures will be developed during the study period, and adapted from the research teams’ previous D&I studies [57].

#### Analysis plan

We anticipate capacity building will significantly improve trainees’ (nurses and community health workers) knowledge and skills in implementation research and delivering of depression intervention. To document feasibility, qualitative training notes and feedback data will be gathered. Themes related to appropriateness and usefulness of the training, partnership and lessons learned for mhGAP/IPT-G implementation capacity building will be analyzed and reported for improvement in future capacity building approach. Quantitative data related to knowledge and competence will be analyzed using pair-t tests and repeated measures to explore impact across different types of MCH personnel.

### Ethical and research protection considerations in working with high risk adolescents

The protection of the rights of research participants is ensured by several aspects of the study protocol:

- First, the principal investigator (PI) and all members of the study team will complete training in human subjects’ protection, as required. This training consists of completion of the formalized training program sponsored by the Kenyan IRB as well as ongoing training by the PI in all aspects of human subjects’ protection through NIH and her mentors’ institutions where she will undergo training.
- Second, all study procedures are reviewed with staff, mentors, and advisory board members from the perspective of ensuring the protection of the rights of study participants. This includes training of study staff in consent and enrollment procedures to minimize coercion and ensure the principles of informed consent, maintaining study material and information in order to protect study participants’ privacy and confidentiality, and ensuring that assessment procedures are conducted in a manner that protects study participants’ privacy and rights. In addition, the study advisory board and Kenyan IRB will inform study participants about protection of their rights by independent institutional board that would have approved the study.
- Third, both study participants and staff will be made aware of the limits to confidentiality. Pregnant adolescents and MCH staff/CHWs will be fully informed of these limits at the time of consent. Study staff will be trained in the requirement to adhere to legal statutes regarding reporting to child protection agencies information obtained in the course of the study that leads a staff member to suspect that an adolescent is at risk for physical abuse or neglect or is being abused or neglected. There is a protocol detailing the responsibility of staff members to share information with the PI immediately or as soon as possible after the information has been obtained. The protocol requires review of the information to determine whether a report to the protection agency is required.
- Finally, in the event of a clinical emergency or other crisis, research staff will be trained in basic crisis management including obstetric and pediatric emergencies and able to contact the PI and her Kenyan mentors. Staff will be trained in a manualized protocol for handling a variety of crises that may present to staff at the health facility or the community venue during interviews. The protocol for handling these issues addresses the safety of both study participants and study staff. The protocol also details the need for supervision in all instances.

#### Integrity of research data

All information collected from study participants during the course of the project will be kept confidentially. This includes information collected and stored in written and electronic form. As indicated previously, data storage also ensures integrity of research data and rights of study participants. Paper copies of identifying information, assessment measures, and other study materials will be maintained in locked research files in locked offices. Electronic data are secured by server maintenance that includes password protection, limited access to data by staff, different levels of access depending on the person’s specific level on the staff, and server securities, all of which ensure a high degree of protection from unauthorized users. Information will be coded by participant identification number. Linking of identifying information to research data will be kept to a minimum and even then, under key and lock and only accessible to key study staff. Identity of study participants will not be revealed in presentations or publications of study findings. Participants will be given the opportunity to consent to (or decline) the use of any specific data collected from them during the intervention phase for use in presentations or publications.

#### Data monitoring

The project staff will continuously evaluate the experience of study participants, with particular regard to participant reactions and risk exposure. Trends in data and findings will be examined periodically (yearly) in order to identify any changes in risk/benefit ratios that might necessitate a modification of the assessment protocol. In the unlikely event that monitoring of data reveals unanticipated or otherwise negative findings; the PI would report these to the field in the form of a presentation or publication. Furthermore, it would be critical to assess, to the degree possible, what characteristics of the study protocol contributed to the findings. Given that the study is nested in a career development grant given to the first author and that it is a pilot trial, the mentors and technical advisory board would act as a data monitoring board along with Fogarty/NIMH annual research reporting. General Purpose of the board: This is not a formal clinical trial DSMB board however a mechanism to address any adverse events that might occur during the study period. Adverse events occurring in any study participants will be reported to the Kenyatta National hospital and University of Nairobi institutional review board within 48 h of occurrence and the external review boards (advisory committee members of the K43 proposal and key mentors at University of Nairobi, University of Washington St Louis and New York University) within 72 h. On a monthly basis the study team will review summary reports of the adverse events. A local Data Safety and Monitoring Board (DSMB) consisting of DW, BA, and OG has been established and will review interim comparisons of the trial arms at 3 and 6 monthly intervals. The general purpose of this board is to maximize the safety and privacy of all study participants, and ensure the integrity, validity, and confidentiality of the data collection and analysis procedures. The protocol described herein details the integration of the goals of the DSMB into all aspects of study design, implementation, and review. The study team will report any serious and unexpected adverse events, or any problems that involve risk to the participants or others, to the Nairobi city council operational research review board and the Kenyan IRB. The study team will report any serious adverse events, along with the Kenyan IRB review response/outcome, to the NIH.

## Discussion

Pregnant adolescents in SSA are burdened by significant unmet health needs including lack of access to mental health services. This study seeks to enrich WHO’s mental health depression intervention by carefully studying potential implementation challenges and identifying and integrating culturally and developmentally sensitive implementation strategies that fit primary care settings in Kenya. This study addresses intervention, service implementation, and public health research gaps for perinatal depression in adolescents in LMICs by utilizing mhGAP-IG, and WHO recommended IPT-G. Considering system, leadership, research strengthening needs (see the description of the Action Plan in Background section) and needs for cost-effective and culturally sensitive depression interventions for pregnant adolescent in Kenya, we will apply a multi-disciplinary/stakeholder partnership approach to adapt and test mhGAP-IG depression intervention guideline/tools and IPT-G to be implemented in MCH clinics. Three key implementation research questions will be studied: (i) how to improve fit of the mhGAP-depression intervention model (including IPT-G) for pregnant adolescents in Kenya, (ii) how to effectively integrate task-shifting/sharing and collaborative strategies with MCH clinical workflow to implement mhGAP depression intervention for pregnant adolescents in MCH settings, and (iii) whether the adapted mhGAP and IPT-G are feasible, appropriate, acceptable, and useful in addressing perinatal depression in adolescents. Considering the demand-resource challenge, this study will also explore related strategies, such as study feasibility of applying Long and Mini version of IPT-G (i.e., 8 vs. 4 sessions) and other potential cost-effective task shifting/sharing strategies (e.g., family/adolescent peer co-lead implementation approach). The IPT-G Full version includes 3 phases of intervention: defining “interpersonal inventory (through examining the interpersonal context of depression and exploring the links between the depression and their environment), making changes (through communication analysis, role play and effective strategies to improve social support and relationships), and identify successes and plan for independent application of IPT principles [41]. It has been found to be highly acceptable and efficacious to perinatal women in LMIC populations [42]. Considering the demand-resource gap, it is critical to consider offering IPT-G Mini version. The development of the shorter (Mini) version will be based on recent work [43, 44] that has developed a three-sessions version. We will adapt and study the fit of the Full and Mini version in MCH contexts (under sub-study 1). After sub-studies 1 and 2 there may be possibilities of reviewing and amending the study protocol to bolster the implementation model and research design for the pilot feasibility trial. In such a scenario, the PI after approvals from the mentors and technical advisory group would make amendments to the IRB.

The proposed research offers following innovative approaches: (1) There is almost no literature on implementation research for adolescent perinatal depression in MCH context using mhGAP evidence-based guidelines. This study tests an innovative collaborative approach that integrates task-shifting/sharing strategies to implement mhGAP guidelines and IPT-G depression intervention in low resource MCH cares in Kenya. (2) Considering Kenyan MCH health service contexts, social cultural contexts, and multiple challenges faced by adolescents, the proposed project explores strategies to address multi-level barriers (especially at the provider, family, and individual levels). Our approach to integrate and test bundled strategies is innovative in addressing multiple challenges that are commonly encountered in Kenya and other LMICs. Thus, the impact of this work may be greater than the traditional EBI efficacy research approach that tends to focus on intervention contents and ignore intervention/service implementation challenges. (3) Our plan to explore Full and Mini versions of IPT-G and other low-cost sustainable strategies (i.e., family empowerment strategies) offers a highly novel alternative solution to address the demand-resource challenge faced in LMICs and many other developed countries. If successful, our study has potential not only to serve more adolescents with lower costs, but also empower communities to contribute to public health solutions. Knowledge gained will be relevant to both LMICs and developed countries.

### Study dissemination plans

Study results would be shared with local communities and agencies through publication channels. Throughout the five-year study period, we will hold several information sharing and dissemination meetings and invite local and international stakeholders (e.g., leaders from Ministry of Health, Nairobi County Health Directorate, other stakeholders such as WHO, UNFPA who have provided support) to discuss adolescent perinatal depression deliberations and trial findings. We will also create brief reports to share findings with relevant local and international health and policy agencies, through multiple publication channels (e.g., University websites, social media, and Fogarty International Centre/NIH, University and interregional and national partnership associated networks). Presenting findings of the work to adolescent groups and MCH young clients in the two informal health centers where the study will be carried out. With the support of the technical and community advisory board we will organize health fair dissemination activities for adolescent groups within the community and MCH centers where peripartum adolescents and other young mothers seek services. Presenting our research study in local and international research and service-related conferences. All final peer-reviewed manuscripts from this study will also be submitted to the digital archive PubMed Central. We will present key findings in the University of Nairobi medical research forums and in the key University research meetings. Information would also be spread through Community Health Unit of Ministry of health and the Nairobi county directorate outreach channels to generate awareness of adolescent pregnancy and mental health burden including available resources to address depression. Progress reports would be submitted to Kenyan partnering agencies and Fogarty Foundation and NIMH Research Office. Each year, the study will prepare a report that summarizes the study’s progress toward recruitment goals, quality of data (e.g., appropriate completion of forms), participation rates, and key findings. Several publications have been planned with the in-country collaborators as well as research mentors.

## Data Availability

NIMH funded research requires the data to be made available for further studies and this would be the case in this work. All the personal information would be de-identified, and the data put on excel sheets for research use. Some interview transcripts would be kept by the researcher and may be shared on reasonable request.

## Abbreviations

WHO: World Health Organization
CFIR: Consolidated Framework for Implementation Research
D&I: Dissemination and implementation
G-IPT/IPT-G: Group interpersonal psychotherapy
MH: Mental health
SDGs: Sustainable development goals
LMICs: Lower- and-middle income countries
KDHS: Kenya demographic health survey
KNBS: Kenya national bureau of statistics
mhGAP-IG: Mental Health Gap Action Program- intervention guide
EBI: Evidence based intervention
MOH: Ministry of Health
MOE: Ministry of Education
UNFPA: United Nations Population Fund
MCH: Maternal and child health
FGD: Focused group interviews
KII: Key informant interviews
NIMH: National Institute of Mental Health, US
